# Supplementary material for: Heparin-based hydrogel scaffolding alters the transcriptomic profile and increases the chemoresistance of MDA-MB-231 triple-negative breast cancer cells
Source: Biomater Sci. 2020 Feb 13;8(10):2786–96. doi: 10.1039/c9bm01481k (PMC7497406; doi:10.1039/c9bm01481k)
Supplement: Supplementary file 2 [file BM-008-C9BM01481K-s002.zip › Supplementary File 4/EGFvControl/Pathways/my_analysis.Gsea.1545200981068/HALLMARK_HEME_METABOLISM.html]

Details for gene set HALLMARK\_HEME\_METABOLISM[GSEA]

|  || Dataset | expr.class.cls#EGF\_versus\_CONTROL.class.cls#EGF\_versus\_CONTROL\_repos |
| Phenotype | class.cls#EGF\_versus\_CONTROL\_repos |
| Upregulated in class | CONTROL |
| GeneSet | HALLMARK\_HEME\_METABOLISM |
| Enrichment Score (ES) | -0.25846106 |
| Normalized Enrichment Score (NES) | -1.2419016 |
| Nominal p-value | 0.048648648 |
| FDR q-value | 0.12474047 |
| FWER p-Value | 0.859 |
Table: GSEA Results Summary

  

Fig 1: Enrichment plot: HALLMARK\_HEME\_METABOLISM      
 Profile of the Running ES Score & Positions of GeneSet Members on the Rank Ordered List

  

| PROBE | DESCRIPTION (from dataset) | GENE SYMBOL | GENE\_TITLE | RANK IN GENE LIST | RANK METRIC SCORE | RUNNING ES | CORE ENRICHMENT || 1 | ABCG2 | na |  |  | 62 | 2.563 | 0.0159 | No |
| 2 | SYNJ1 | na |  |  | 277 | 1.980 | 0.0195 | No |
| 3 | MAP2K3 | na |  |  | 282 | 1.978 | 0.0341 | No |
| 4 | ABCB6 | na |  |  | 292 | 1.964 | 0.0484 | No |
| 5 | PQLC1 | na |  |  | 350 | 1.888 | 0.0596 | No |
| 6 | TFRC | na |  |  | 359 | 1.882 | 0.0732 | No |
| 7 | PSMD9 | na |  |  | 480 | 1.782 | 0.0803 | No |
| 8 | TNS1 | na |  |  | 617 | 1.692 | 0.0858 | No |
| 9 | RCL1 | na |  |  | 820 | 1.581 | 0.0870 | No |
| 10 | MPP1 | na |  |  | 959 | 1.525 | 0.0912 | No |
| 11 | GCLM | na |  |  | 1109 | 1.475 | 0.0944 | No |
| 12 | E2F2 | na |  |  | 1450 | 1.364 | 0.0868 | No |
| 13 | UCP2 | na |  |  | 1461 | 1.359 | 0.0965 | No |
| 14 | SLC7A11 | na |  |  | 1471 | 1.356 | 0.1061 | No |
| 15 | BMP2K | na |  |  | 1665 | 1.304 | 0.1058 | No |
| 16 | FBXO9 | na |  |  | 1928 | 1.240 | 0.1013 | No |
| 17 | XK | na |  |  | 2117 | 1.194 | 0.1004 | No |
| 18 | CCND3 | na |  |  | 2241 | 1.169 | 0.1027 | No |
| 19 | NEK7 | na |  |  | 2286 | 1.159 | 0.1090 | No |
| 20 | GMPS | na |  |  | 2348 | 1.145 | 0.1144 | No |
| 21 | RBM38 | na |  |  | 2349 | 1.145 | 0.1230 | No |
| 22 | SLC25A38 | na |  |  | 2738 | 1.070 | 0.1106 | No |
| 23 | FECH | na |  |  | 2757 | 1.067 | 0.1177 | No |
| 24 | USP15 | na |  |  | 3028 | 1.022 | 0.1112 | No |
| 25 | HIST1H4C | na |  |  | 3214 | 0.984 | 0.1088 | No |
| 26 | OSBP2 | na |  |  | 3308 | 0.969 | 0.1112 | No |
| 27 | TRAK2 | na |  |  | 3469 | 0.942 | 0.1098 | No |
| 28 | UROS | na |  |  | 3873 | 0.873 | 0.0952 | No |
| 29 | TSPAN5 | na |  |  | 3909 | 0.866 | 0.0999 | No |
| 30 | ELL2 | na |  |  | 4025 | 0.847 | 0.1002 | No |
| 31 | NNT | na |  |  | 4026 | 0.847 | 0.1065 | No |
| 32 | AGPAT4 | na |  |  | 4103 | 0.836 | 0.1088 | No |
| 33 | DCUN1D1 | na |  |  | 4244 | 0.816 | 0.1075 | No |
| 34 | SEC14L1 | na |  |  | 4386 | 0.794 | 0.1061 | No |
| 35 | HMBS | na |  |  | 4409 | 0.790 | 0.1108 | No |
| 36 | GAPVD1 | na |  |  | 4453 | 0.784 | 0.1145 | No |
| 37 | TMEM9B | na |  |  | 4578 | 0.767 | 0.1137 | No |
| 38 | RBM5 | na |  |  | 4691 | 0.750 | 0.1134 | No |
| 39 | SLC30A1 | na |  |  | 4777 | 0.734 | 0.1145 | No |
| 40 | TCEA1 | na |  |  | 4826 | 0.725 | 0.1174 | No |
| 41 | NR3C1 | na |  |  | 5093 | 0.695 | 0.1086 | No |
| 42 | CROCCP2 | na |  |  | 5260 | 0.670 | 0.1049 | No |
| 43 | ISCA1 | na |  |  | 5371 | 0.654 | 0.1040 | No |
| 44 | ARHGEF12 | na |  |  | 5421 | 0.647 | 0.1063 | No |
| 45 | XPO7 | na |  |  | 5579 | 0.628 | 0.1028 | No |
| 46 | RHD | na |  |  | 6204 | 0.541 | 0.0740 | No |
| 47 | DAAM1 | na |  |  | 6318 | 0.524 | 0.0720 | No |
| 48 | MOSPD1 | na |  |  | 6390 | 0.515 | 0.0722 | No |
| 49 | GCLC | na |  |  | 6772 | 0.472 | 0.0557 | No |
| 50 | MFHAS1 | na |  |  | 6867 | 0.460 | 0.0542 | No |
| 51 | ADIPOR1 | na |  |  | 7028 | 0.443 | 0.0491 | No |
| 52 | FBXO34 | na |  |  | 7033 | 0.443 | 0.0522 | No |
| 53 | PICALM | na |  |  | 7165 | 0.426 | 0.0485 | No |
| 54 | ATP6V0A1 | na |  |  | 7208 | 0.421 | 0.0495 | No |
| 55 | CTSE | na |  |  | 7387 | 0.398 | 0.0431 | No |
| 56 | NCOA4 | na |  |  | 7590 | 0.374 | 0.0353 | No |
| 57 | ANK1 | na |  |  | 7893 | 0.337 | 0.0219 | No |
| 58 | UROD | na |  |  | 7917 | 0.336 | 0.0232 | No |
| 59 | CDR2 | na |  |  | 7946 | 0.332 | 0.0243 | No |
| 60 | CDC27 | na |  |  | 7997 | 0.325 | 0.0241 | No |
| 61 | FBXO7 | na |  |  | 8134 | 0.314 | 0.0193 | No |
| 62 | ATG4A | na |  |  | 8138 | 0.313 | 0.0215 | No |
| 63 | RANBP10 | na |  |  | 8171 | 0.308 | 0.0221 | No |
| 64 | MOCOS | na |  |  | 8249 | 0.299 | 0.0203 | No |
| 65 | RNF19A | na |  |  | 8418 | 0.282 | 0.0136 | No |
| 66 | KAT2B | na |  |  | 8458 | 0.278 | 0.0136 | No |
| 67 | BTRC | na |  |  | 8667 | 0.252 | 0.0046 | No |
| 68 | GDE1 | na |  |  | 8739 | 0.241 | 0.0026 | No |
| 69 | MBOAT2 | na |  |  | 8768 | 0.240 | 0.0030 | No |
| 70 | HTATIP2 | na |  |  | 8827 | 0.234 | 0.0017 | No |
| 71 | FOXO3 | na |  |  | 9106 | 0.201 | -0.0114 | No |
| 72 | MARK3 | na |  |  | 9342 | 0.179 | -0.0224 | No |
| 73 | UBAC1 | na |  |  | 9421 | 0.167 | -0.0253 | No |
| 74 | ALDH1L1 | na |  |  | 9719 | 0.135 | -0.0399 | No |
| 75 | LMO2 | na |  |  | 9839 | 0.122 | -0.0452 | No |
| 76 | RAP1GAP | na |  |  | 9901 | 0.116 | -0.0475 | No |
| 77 | HTRA2 | na |  |  | 9952 | 0.108 | -0.0494 | No |
| 78 | GLRX5 | na |  |  | 10174 | 0.085 | -0.0603 | No |
| 79 | ENDOD1 | na |  |  | 10248 | 0.075 | -0.0636 | No |
| 80 | LAMP2 | na |  |  | 11032 | -0.007 | -0.1047 | No |
| 81 | HEBP1 | na |  |  | 11138 | -0.018 | -0.1101 | No |
| 82 | TFDP2 | na |  |  | 11153 | -0.021 | -0.1107 | No |
| 83 | PPOX | na |  |  | 11168 | -0.022 | -0.1112 | No |
| 84 | CPOX | na |  |  | 11388 | -0.048 | -0.1224 | No |
| 85 | RIOK3 | na |  |  | 11696 | -0.085 | -0.1379 | No |
| 86 | TOP1 | na |  |  | 11743 | -0.091 | -0.1396 | No |
| 87 | SLC22A4 | na |  |  | 11928 | -0.117 | -0.1484 | No |
| 88 | BTG2 | na |  |  | 11938 | -0.118 | -0.1480 | No |
| 89 | SPTB | na |  |  | 12076 | -0.136 | -0.1542 | No |
| 90 | EIF2AK1 | na |  |  | 12432 | -0.175 | -0.1715 | No |
| 91 | ADD1 | na |  |  | 12475 | -0.181 | -0.1724 | No |
| 92 | ASNS | na |  |  | 12529 | -0.188 | -0.1737 | No |
| 93 | BPGM | na |  |  | 12551 | -0.191 | -0.1734 | No |
| 94 | SLC2A1 | na |  |  | 12606 | -0.200 | -0.1747 | No |
| 95 | PPP2R5B | na |  |  | 12652 | -0.205 | -0.1756 | No |
| 96 | DCAF10 | na |  |  | 12744 | -0.221 | -0.1787 | No |
| 97 | PIGQ | na |  |  | 12903 | -0.231 | -0.1853 | No |
| 98 | MKRN1 | na |  |  | 13184 | -0.271 | -0.1979 | No |
| 99 | HAGH | na |  |  | 13185 | -0.271 | -0.1959 | No |
| 100 | SLC10A3 | na |  |  | 13226 | -0.277 | -0.1959 | No |
| 101 | HBQ1 | na |  |  | 13285 | -0.287 | -0.1968 | No |
| 102 | TNRC6B | na |  |  | 13460 | -0.308 | -0.2037 | No |
| 103 | SLC11A2 | na |  |  | 13608 | -0.331 | -0.2089 | No |
| 104 | DCAF11 | na |  |  | 13711 | -0.347 | -0.2117 | No |
| 105 | PGLS | na |  |  | 13995 | -0.377 | -0.2237 | No |
| 106 | RAD23A | na |  |  | 14155 | -0.399 | -0.2291 | No |
| 107 | BLVRA | na |  |  | 14181 | -0.403 | -0.2274 | No |
| 108 | EPB41 | na |  |  | 14182 | -0.403 | -0.2243 | No |
| 109 | SMOX | na |  |  | 14262 | -0.416 | -0.2254 | No |
| 110 | BLVRB | na |  |  | 14274 | -0.418 | -0.2228 | No |
| 111 | VEZF1 | na |  |  | 14398 | -0.433 | -0.2260 | No |
| 112 | FOXJ2 | na |  |  | 14399 | -0.433 | -0.2228 | No |
| 113 | HDGF | na |  |  | 14471 | -0.440 | -0.2232 | No |
| 114 | CTNS | na |  |  | 14495 | -0.444 | -0.2211 | No |
| 115 | SDCBP | na |  |  | 14589 | -0.461 | -0.2225 | No |
| 116 | NFE2 | na |  |  | 14720 | -0.484 | -0.2257 | No |
| 117 | TSPO2 | na |  |  | 14845 | -0.502 | -0.2285 | No |
| 118 | RNF123 | na |  |  | 15278 | -0.560 | -0.2470 | No |
| 119 | MARCH2 | na |  |  | 15340 | -0.573 | -0.2459 | No |
| 120 | KHNYN | na |  |  | 15485 | -0.594 | -0.2490 | No |
| 121 | ARL2BP | na |  |  | 15550 | -0.602 | -0.2479 | No |
| 122 | NUDT4 | na |  |  | 15620 | -0.609 | -0.2469 | No |
| 123 | CAT | na |  |  | 15662 | -0.617 | -0.2445 | No |
| 124 | CLCN3 | na |  |  | 15758 | -0.639 | -0.2447 | No |
| 125 | SIDT2 | na |  |  | 15962 | -0.679 | -0.2502 | No |
| 126 | MINPP1 | na |  |  | 16105 | -0.705 | -0.2524 | No |
| 127 | H1F0 | na |  |  | 16221 | -0.737 | -0.2529 | Yes |
| 128 | FN3K | na |  |  | 16272 | -0.751 | -0.2499 | Yes |
| 129 | NFE2L1 | na |  |  | 16329 | -0.764 | -0.2472 | Yes |
| 130 | CAST | na |  |  | 16336 | -0.765 | -0.2417 | Yes |
| 131 | PRDX2 | na |  |  | 16340 | -0.766 | -0.2362 | Yes |
| 132 | YPEL5 | na |  |  | 16491 | -0.809 | -0.2380 | Yes |
| 133 | BSG | na |  |  | 16641 | -0.844 | -0.2395 | Yes |
| 134 | EZH1 | na |  |  | 16657 | -0.848 | -0.2339 | Yes |
| 135 | MARCH8 | na |  |  | 16673 | -0.851 | -0.2283 | Yes |
| 136 | FAM46C | na |  |  | 16752 | -0.870 | -0.2259 | Yes |
| 137 | BACH1 | na |  |  | 17003 | -0.952 | -0.2319 | Yes |
| 138 | IGSF3 | na |  |  | 17102 | -0.976 | -0.2297 | Yes |
| 139 | ALDH6A1 | na |  |  | 17199 | -1.008 | -0.2272 | Yes |
| 140 | SLC6A9 | na |  |  | 17329 | -1.052 | -0.2261 | Yes |
| 141 | PDZK1IP1 | na |  |  | 17332 | -1.053 | -0.2183 | Yes |
| 142 | PC | na |  |  | 17387 | -1.072 | -0.2131 | Yes |
| 143 | BNIP3L | na |  |  | 17581 | -1.142 | -0.2147 | Yes |
| 144 | C3 | na |  |  | 17617 | -1.156 | -0.2079 | Yes |
| 145 | TMCC2 | na |  |  | 17645 | -1.165 | -0.2006 | Yes |
| 146 | LRP10 | na |  |  | 17662 | -1.172 | -0.1926 | Yes |
| 147 | AQP3 | na |  |  | 17703 | -1.187 | -0.1859 | Yes |
| 148 | MGST3 | na |  |  | 17776 | -1.214 | -0.1805 | Yes |
| 149 | KLF3 | na |  |  | 17901 | -1.275 | -0.1775 | Yes |
| 150 | SLC6A8 | na |  |  | 17923 | -1.288 | -0.1690 | Yes |
| 151 | OPTN | na |  |  | 17950 | -1.301 | -0.1606 | Yes |
| 152 | P4HA2 | na |  |  | 17972 | -1.316 | -0.1518 | Yes |
| 153 | CIR1 | na |  |  | 17989 | -1.323 | -0.1427 | Yes |
| 154 | ALAD | na |  |  | 18280 | -1.479 | -0.1469 | Yes |
| 155 | EPOR | na |  |  | 18353 | -1.528 | -0.1392 | Yes |
| 156 | LPIN2 | na |  |  | 18431 | -1.604 | -0.1313 | Yes |
| 157 | NARF | na |  |  | 18628 | -1.783 | -0.1282 | Yes |
| 158 | CTSB | na |  |  | 18701 | -1.896 | -0.1178 | Yes |
| 159 | SLC30A10 | na |  |  | 18706 | -1.908 | -0.1037 | Yes |
| 160 | SLC25A37 | na |  |  | 18797 | -2.057 | -0.0930 | Yes |
| 161 | BCAM | na |  |  | 18802 | -2.079 | -0.0776 | Yes |
| 162 | ERMAP | na |  |  | 18815 | -2.099 | -0.0625 | Yes |
| 163 | CCDC28A | na |  |  | 18827 | -2.122 | -0.0472 | Yes |
| 164 | SELENBP1 | na |  |  | 18959 | -2.473 | -0.0356 | Yes |
| 165 | MXI1 | na |  |  | 19008 | -2.689 | -0.0179 | Yes |
| 166 | CLIC2 | na |  |  | 19151 | -3.717 | 0.0025 | Yes |
Table: GSEA details [plain text format]

  

Fig 2: HALLMARK\_HEME\_METABOLISM      
 Blue-Pink O' Gram in the Space of the Analyzed GeneSet

  

Fig 3: HALLMARK\_HEME\_METABOLISM: Random ES distribution      
 Gene set null distribution of ES for **HALLMARK\_HEME\_METABOLISM**
